# Light alters calling-song characteristics in crickets

**DOI:** 10.1242/jeb.249404

**Published:** 2025-02-25

**Authors:** Keren Levy, Yossef Yits'hak Aidan, Dror Paz, Heba Medlij, Amir Ayali

**Affiliations:** ^1^ School of Zoology, Tel Aviv University, Tel-Aviv 6997801, Israel; ^2^ Sagol School of Neuroscience, Tel Aviv University, Tel-Aviv 6997801, Israel

**Keywords:** Bioacoustics, *Gryllus bimaculatus*, Sexual selection, Intraspecific communication, Stridulation, Computational analysis

## Abstract

Communication is crucial for mate choice and thus for the survival and fitness of most species. In the cricket, females choose males according to their calling-song attractiveness and, exhibiting positive phonotaxis, they approach the chosen male. Light has been widely reported to induce changes in crickets' daily activity patterns, including the males' stridulation behavior. It had remained unknown, however, whether light also affects the calling-song properties and thus may consequently also alter female choice. Here, we present a novel semi-automated process, enabling the analysis of calling-song properties in an extremely large sample size of recording sections from males subjected to lifelong light:dark (LD) or constant light (LL) conditions. Our findings revealed that the LD calling songs consisted of longer chirps, longer inter-syllable intervals and a higher proportion of 4-syllable chirps compared with those of LL males. We also conducted some preliminary female choice experiments suggesting that females (reared in LD conditions) exposed to playbacks of male calling songs exhibit a preference towards LD over LL recordings. We therefore conclude that illumination conditions such as constant light affect the male crickets' calling-song properties in a manner that may be discernible to the females. It remains unclear, however, how and to what extent female mate choice and the species' overall fitness are affected by these changes.

## Teaser

**Summary:** An in-depth analysis of a large-scale dataset of male cricket calling songs, utilizing a novel tool for semi-automated detection of temporal characteristics, reveals that illumination conditions affect calling-song properties.

## INTRODUCTION

Cricket courtship behavior is tightly coupled to the day–night cycle, usually starting at sunset and persisting throughout the night, thus presenting a clear circadian pattern (Fabre et al., 1921; Loher, 1989). Overall, the exposure of living organisms to light and darkness serves as a circadian cue for internal timekeeping and synchronizes the animal's temporal activity to its environment (Daan and Aschoff, 2001). This includes the timing of sexual maturation, as well as courtship behavior. A century of research has revealed various impacts of light on the crickets' circadian rhythms (Kutaragi et al., 2018; Levy et al., 2024a; Numata and Tomioka, 2023; Tomioka and Chiba, 1982), including on their timing of locomotion and stridulation activity (Huber et al., 1989; Levy et al., 2021, 2024a; Sokolove, 1975), on intraspecific communication, as well as on gene expression (Levy et al., 2022; Moriyama et al., 2009; Numata and Tomioka, 2023). However, it remains unknown whether light or illumination patterns also affect the distinct properties of the crickets' calling-song components, and thus possibly also mate choice.

Intraspecific communication is vital for the reproductive success of a species and depends on the reliability of the transfer of information about the sender to the receiver, as well as on the precise timing of their interaction (Greenfield, 2002; Loher and Dambach, 1989; Nityananda and Balakrishnan, 2021; Tauber et al., 2001). In crickets, this involves all the sensory modalities, including visual, acoustic, chemical (pheromones) and tactile stimuli (Greenfield, 2002; Horch et al., 2017; Simmons, 1988; ter Hofstede et al., 2015), while the acoustic signals serve for species-specific communication such as in courtship behavior (Alexander, 1960; Loher and Dambach, 1989).

**Fig. 1.**
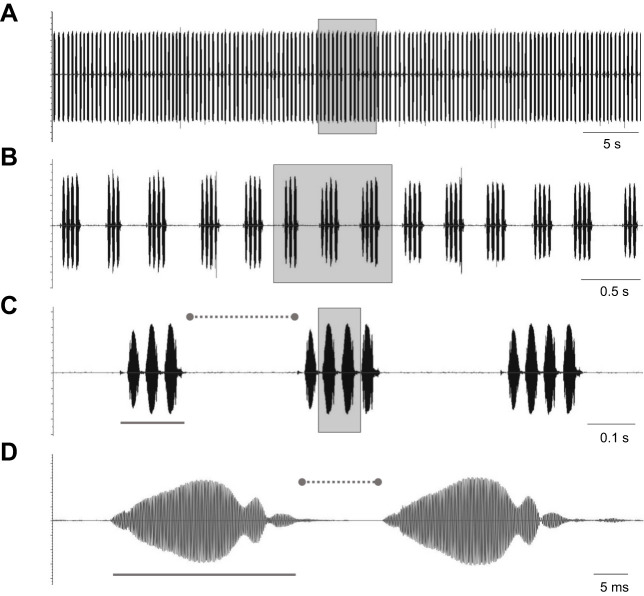
**Time series representation of the *Gryllus bimaculatus* male cricket stridulation.** (A) The crickets' calling song consists of chirps and inter-chirp intervals. (B) An enlarged view of the shaded square from A, showing that each chirp comprises 3 or 4 syllables. (C) An enlarged view of the shaded square from B: the solid bar denotes one chirp; the dotted bar denotes the inter-chirp interval. (D) An enlarged view of the shaded square from C: the solid bar indicates one syllable; the dotted bar denotes the inter-syllable interval.

In the field cricket (*Gryllus* sp.), males demonstrate stridulation, producing distinct calling songs (Fig. 1), while females exhibit precise, positive phonotaxis towards this acoustic signal (Regen, 1913; Schöneich and Hedwig, 2010). The males stridulate by rapidly rubbing their forewings (tegmina) together. The closing movement of the tegmina includes the movement of the file (made of a tooth row, located on the right wing) over the plectrum (on the left wing), thus producing sound (Bennet-Clark, 1999; Huber, 1962; Montealegre-Z et al., 2009). In *Gryllus bimaculatus* crickets, the calling song is composed of repeated chirps, each containing several syllables (also frequently referred to as ‘pulses’; Fig. 1), with chirps consisting of 3 or 4 syllables being the most prevalent (Doherty, 1985; Lin and Hedwig, 2021; Popov and Shuvalov, 1977).

The females are generally attracted by, and hence base their mate choice on, the distinct characteristics of the male calling song (followed by courtship songs and mutual antennation) (Alexander, 1960; Hall and Robinson, 2021; Loher and Dambach, 1989; Schmidt et al., 2014). The acoustic orientation of *G. bimaculatus* females has been reported to be one of the most precise among invertebrates (Schöneich and Hedwig, 2010). The most intense female phonotaxis behavior is demonstrated at the age of 7–24 days following adult eclosion, which is also correlated with the female's highest egg production stage (Sarmiento-Ponce et al., 2021). Studies of auditory brain neurons in crickets indicate that the syllable period and the intervals between syllables are crucial for species-specific recognition (Popov et al., 1974; Schöneich et al., 2015). Furthermore, certain characteristics of the song, including carrier frequency, inter-syllable interval, chirp rate and call intensity, are believed to be influenced by sexual selection because of their correlation with the male traits preferred by females, such as body size and age, which indicate male quality (Ferreira and Ferguson, 2002; Grobe et al., 2012; Judge, 2011; Kuriwada, 2023; Simmons, 1988; Verburgt et al., 2011; Zhemchuzhnikov et al., 2017; Zhemchuzhnikov and Knyazev, 2015). Another component that may affect female mate choice is that of the females' growth conditions and nutrition (Judge et al., 2014).

In addition to a major effect of the crickets' genetics, both biotic and abiotic environmental factors, prior to as well as during the mate-finding behavior, are important factors responsible for intraspecific variation and plasticity in the males' calling song and the females' mate choice preferences (Beckers et al., 2019; Walker, 1962). One such environmental factor is temperature, which has been reported to affect both male stridulation frequency (Beckers, 2020; Doherty, 1985; Dolbear, 1897; Levy et al., 2024a,b) and female mate choice preferences (Beckers et al., 2019; Symes et al., 2017), thus impacting mating preferences.

We have previously investigated in depth the effects of artificial light at night (ALAN) on the timing and synchronization of field crickets' stridulation behavior. We reported that changes in lighting conditions affect the crickets' circadian behavior, reflected in changes in the individual's timing of stridulation and locomotion activity, both in the laboratory and under almost natural conditions (Levy et al., 2021, 2024b), hence affecting intraspecific communication. However, whether illumination conditions also affect any of the calling-song properties and thus possibly also mate choice remained unknown to date.

Here, we studied for the first time the effects of lifelong exposure to different light conditions on the calling-song properties of male *G. bimaculatus* crickets. We used the vast set of cricket calling-song recordings obtained in our previous laboratory study (Levy et al., 2021), which comprised two extreme illumination conditions: light–dark and constant light regimes, and tested possible illumination-induced changes in the crickets' calling-song properties*.* Additionally, we developed a novel semi-automated process that enables the analysis of calling-song properties, using an extremely large sample size of recording sections. Finally, a set of preliminary experiments suggests that the females' mate choices may be affected by the males' illumination conditions.

## MATERIALS AND METHODS

### Rearing conditions

*Gryllus bimaculatus* De Geer 1773 crickets were reared in the laboratory under a constant temperature of 26±2°C in a chamber illuminated with white compact fluorescent light (CFL, NeptOn, 6500K, 380–780 nm, peaks: 547 and 612 nm). They were fed three times a week with dog chow and vegetables. The rearing boxes contained water flasks with absorbent cotton wool and egg cartons for shelter.

The experimental male crickets were exposed from the egg stage throughout all life stages, including the adult stage (lifelong, as described in Levy et al., 2021), to one of two illumination conditions: either control light conditions of diurnal 12 h light of 40 lx and nocturnal 12 h dark (LD) or 24 h of constant light of 40 lx (LL).

### Calling-song recordings

Sexually mature male crickets were isolated, individually housed in an anechoic chamber and their stridulation was recorded for a minimum of five consecutive days and nights (for further details, see Levy et al., 2021). These recordings constitute our library of calling songs, providing the recordings used here for the sonogram analysis as well as for the female choice playback experiments. Each recording was of 30 min duration and contained a minimum of 10 min of male stridulation.

### Full data analysis of chirp duration

A full data analysis was conducted on our entire calling-song library (Levy et al., 2021). Chirp duration analysis was performed by extracting all stridulation events from all individuals using ‘R’, version 3.4.1 (http://www.R-project.org/), the ‘Rraven’ open source package (https://cran.r-project.org/web/packages/warbleR/index.html) and the ‘Band limited energy detector’ function in RavenPro 1.5. Only data from individuals recorded for more than 4 days and containing more than 20,000 chirps were included.

### Calling-song parameter analysis

For an in-depth analysis, the male cricket calling song recordings of randomly chosen 5 LD and 5 LL individuals were analyzed (5 recordings from each, a total of 50). Song properties to be compared included overall frequency, chirp count, chirp duration and interval, and the proportion of 3-syllable versus 4-syllable chirps. In order to conduct a more detailed analysis, 3500 chirps were obtained from each of the above recordings. Data comprised in-depth properties comparison of 3-syllable versus 4-syllable chirps, including the syllable and inter-syllable durations.

**Fig. 2.**
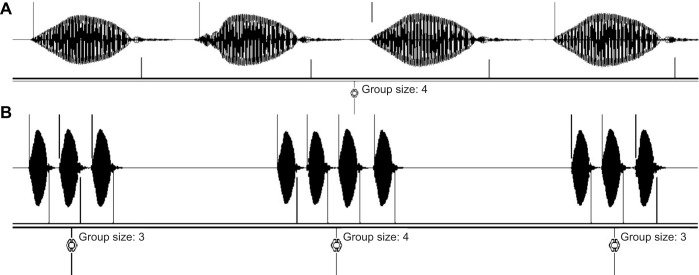
**A screen capture of the time series representation of the automated detection of a male cricket's calling-song properties.** (A) Detection of the onset and offset of syllables and (B) their grouping into 3- and 4-syllable chirps. The vertical lines at the onset and offset points of each syllable provide visual validation of the automated detection process.

Calling song analysis was conducted using the Python programming language (Libroas Library, version 3.10, PyCharm, JetBrains) and Audacity^®^ Sound Editor (v.3.2.1; https://www.audacityteam.org/). First, the audio signal amplitudes were normalized to a range of −1 to 1 and the amplitude envelope was extracted. The analyses were carried out using many small fragments of the audio recording. The crickets' calling-song events were defined as values exceeding a 0.1 threshold, and a second, low, threshold was applied to eliminate noise between chirps and syllables. For each syllable and chirp, the onset and offset border (Fig. 2) was defined at zero amplitude. The Audacity editor was used to visually inspect and validate the automated detection (Fig. 2) and its accuracy. The number of syllables for each chirp was then annotated (Fig. 2B). The inter-syllable and inter-chirp intervals were detected similarly, using a minimum and maximum duration threshold to differentiate them from other pauses in the calling song.

This novel custom-developed method enabled us to accurately delineate the borders of syllables and chirps within the audio signal, enabling an automated and detailed extraction and analysis of the crickets' calling songs, followed by a comparison between treatments. The code for the semi-automated detection is available to download from GitHub (https://github.com/Time2bImmortal/Concert_of_the_night.git).

### Statistical analysis

Data processing and statistical analyses were conducted using Python v. 3.10, SPSS v. 21 (IBM Corp., Armonk, NY, USA) and Prism 8 (GraphPad Software, San Diego, CA, USA). A Welch *t*-test was used to compare the mean chirp duration for the overall full-data analysis, and the *F*-test was used to test for equality of variance. For the sonogram analysis, each sound file for the semi-automated calling-song analysis contained at least 3500 chirps. The frequency, duration of chirp and inter-chirp interval were averaged for the overall recordings, and the syllable and inter-syllable interval durations were averaged for 3500 chirps per recording. All calling-song parameters (chirp and syllable count, chirp, syllable and their interval durations, as well as the overall frequency) were then tested for normality, if needed transformed to achieve normal distribution (Table S1), and followed by a nested *t*-test comparing the two treatments (LD and LL). The proportions of the number of syllables per chirp were analyzed using the χ^2^ test comparing the observed versus expected proportions.

## RESULTS

### Full-data analysis of chirp duration

**Fig. 3.**
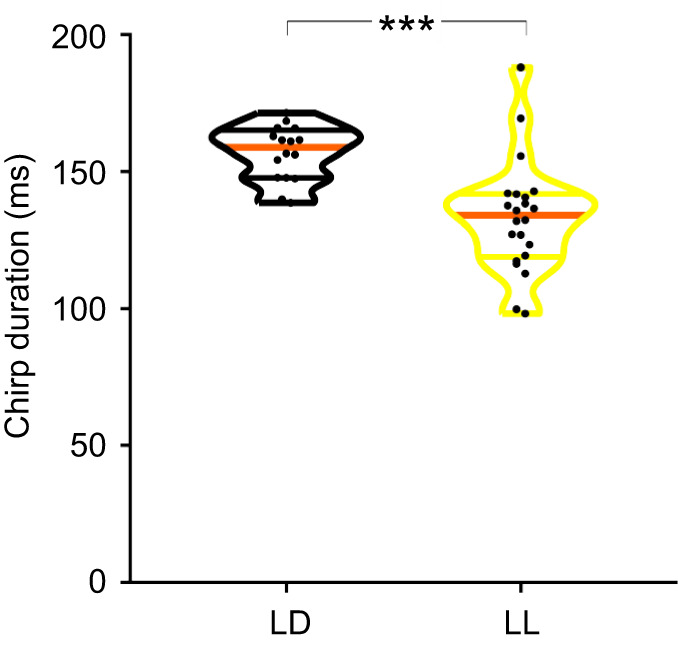
**Lifelong exposure to constant light affects calling-song properties of *G. bimaculatus* males, expressed as a decrease in the median chirp duration and increased variance.** Medians are colored orange; the average stridulation of each individual male cricket is represented by a dot. Light:dark (LD), *n=*16; constant light (LL), *n=*22. Asterisks indicate significance (Welch *t*-test: ***P<0.001).

To investigate any possible differences in chirp duration between LD and LL groups, the overall mean chirp duration was calculated for all the relevant data extracted in Levy et al. (2021) using RavenPro 1.5 for the detection. The male's calling song mean chirp duration was found to be affected by the exposure to lifelong illumination conditions. A significant decrease in chirp duration was observed under LL compared with LD conditions (*n*_LD_=16 insects; *n*_LL_=22; Welch *t*-test, *P<*0.001; Fig. 3). Furthermore, the variance in chirp duration significantly differed between LD and LL treatments (*F*-test, *P=*0.006; Fig. 3).

### Calling-song properties analysis

**Fig. 4.**
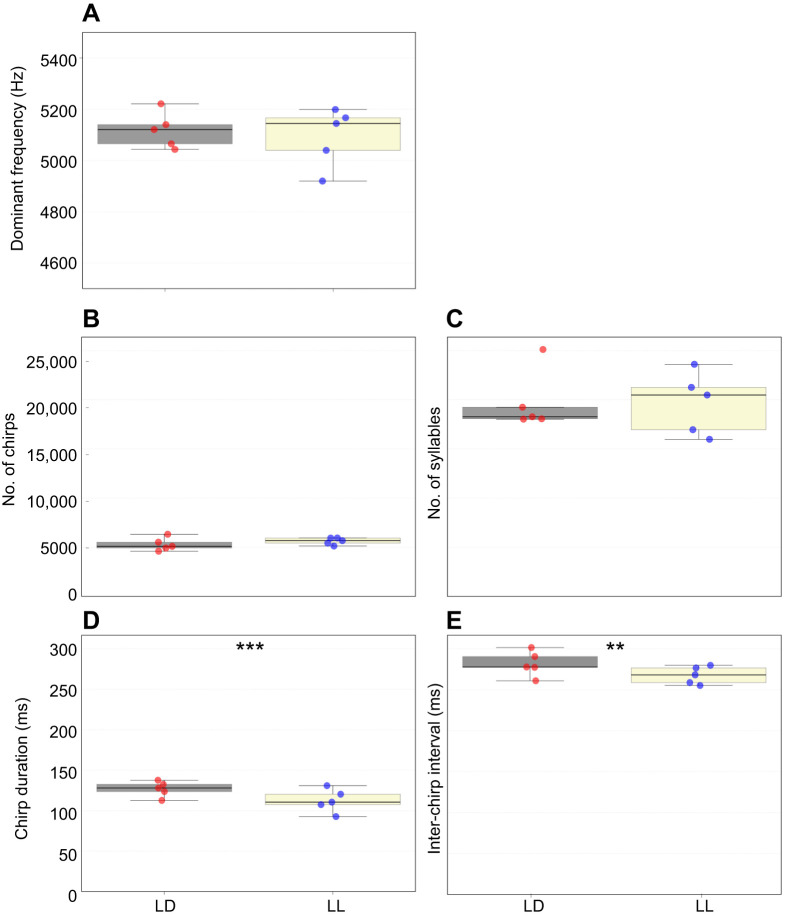
**Calling-song properties of male crickets reared under lifelong LD or LL lighting conditions.** The dominant frequency (A), mean number of chirps (B) and mean number of syllables (C) did not differ significantly between the two lighting conditions. However, both the mean chirp duration (D) and the mean inter-chirp interval duration (E) differed significantly between LD and LL (nested *t*-test: **P<0.01, ***P<0.001).

Further examination of the calling-song properties in large sections of the LD and LL recordings was conducted using our custom-developed code, revealing several light-related differences. As noted, the crickets' calling song is composed of chirps, each consisting of syllables (Fig. 1). While the dominant frequency and both the overall number of chirps and the overall number of syllables were similar between treatments (Fig. 4A–C; nested *t*-test, *P*>0.1), the mean chirp duration was found to be significantly longer in LD calling songs than in LL calling songs (Fig. 4D; nested *t*-test, *P*=0.005). Also, the mean duration of the inter-chirp interval (pauses between chirps) was found to be significantly shorter under LL conditions compared with LD conditions (Fig. 4E; nested *t*-test, *P*<0.004).

Some discrepancy in the mean chirp duration presented in Figs 3 and 4 arises from differences in the algorithms used by the two analysis tools. Specifically, these differences result from the range each program considers as part of the signal before and after the chirp: RavenPro 1.5 applies a larger chirp range compared with the semi-automated detection method. Importantly, a consistent light-induced difference is revealed irrespective of the utilized analyses tool.

Interestingly, when comparing the mean inter-chirp intervals, the interval at the transition between 3- to 4-syllables chirps was found to be significantly longer in LD than in LL calling song (Table S2). Data for the reverse transition, 4- to 3-syllables chirps, were very limited or lacking altogether. It should also be noted that in a few cases, the LL recordings consisted of 3-syllables chirps only (lacking 4-syllable chirps altogether).

**Fig. 5.**
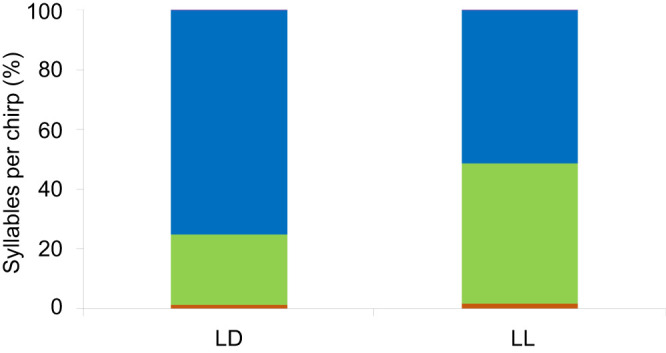
**Distribution and proportion of 1-, 2-, 3-, 4- and 5-syllable chirps in the calling-song recordings of male crickets reared under lifelong LD or LL conditions.** The 3- and 4-syllable chirps (green and blue, respectively) are the most dominant, comprising >98% of the total (LD: 23.54% and 75.07%; LL: 46.99% and 51.17%, respectively); 2-syllable chirps (red) comprise <2% of the total. In both LD and LL, 1- or 5-syllable chirps were practically absent.

The number of syllables per chirp in the calling song can vary between 1 and 5 (and up to 12 in aggressive signaling), with 3- and 4-syllable chirps being the most prevalent (their cumulative proportion exceeded 98% in both LD and LL recordings). The noted difference in chirp duration could thus result from a difference in the number of syllables per chirp, i.e. a different proportion of 4- versus 3-syllable chirps in calling songs of the two experimental groups. Indeed, the proportions of the number of syllables per chirp evaluated for the LD and LL calling songs revealed significant differences between the observed and a random distribution in both LD and LL (Chi-square test; χ_4,5000_=10,476, *P*<0.001 and χ_4,5000_=7071, *P*<0.001, respectively; Fig. 5). The proportion of 3- and 4-syllable chirps significantly differed from a 50:50 distribution within each treatment and between the two treatments (Chi-square test; χ_1,4931_=1346, *P*<0.001 and χ_1,4908_=8.82, *P*<0.003 for LD and LL, respectively). A significantly higher proportion of 4-syllable chirps in LD (nested *t*-test; 75.07% and 51.17% for LD and LL, respectively; *P*=0.043) compared with a higher proportion of 3-syllable chirps in LL (nested *t*-test; 23.54% and 46.99% for LD and LL, respectively; *P*=0.045) was observed (Fig. 5), thereby demonstrating a light-induced effect on this calling-song parameter.

### Calling-song parameter analysis: inter-syllable intervals differ between treatments

**Fig. 6.**
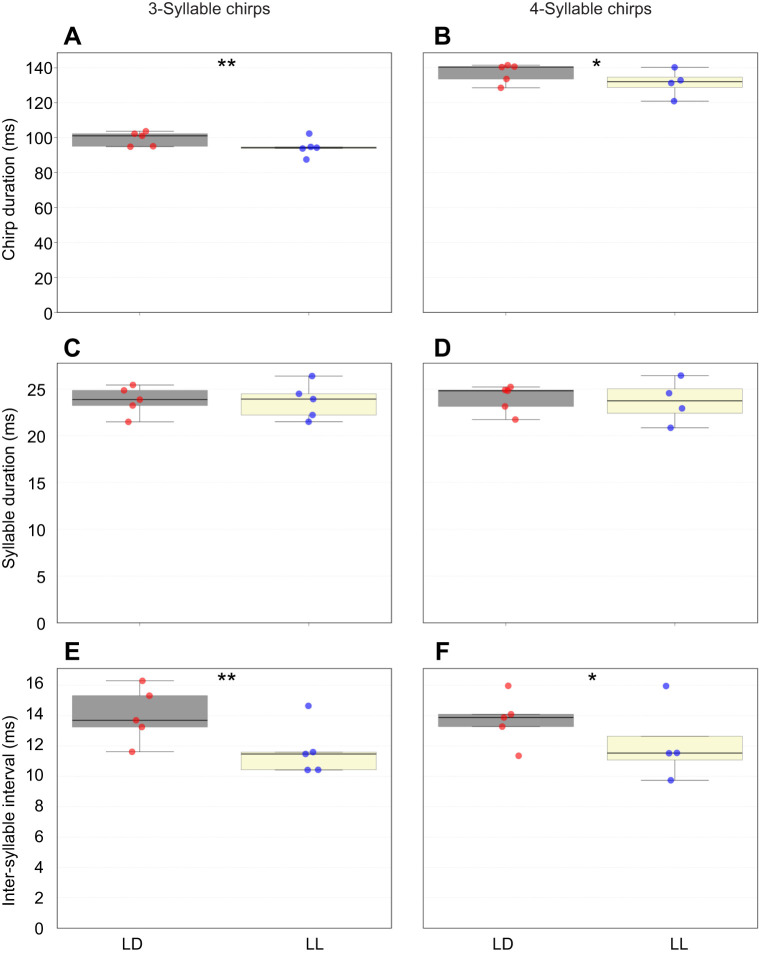
**Calling-song properties of male crickets reared under lifelong LD or LL illumination.** A total of 3500 chirps were analyzed from each of five sound recordings for each of the five experimental crickets per treatment. Lifelong lighting conditions significantly affected 3-syllable (A) and 4-syllable (B) chirp duration. It did not affect syllable duration for either 3-syllable (C) or 4-syllable chirps (D). However, lifelong constant illumination did significantly affect the inter-syllable interval duration in both 3-syllable (E) and 4-syllable (F) chirps. Asterisks indicate significance (nested *t*-test: *P<0.05, **P<0.01).

Although we established a difference in the proportion of 3- versus 4-syllable chirps between the calling songs of the two experimental groups (Fig. 5), the observed difference in the mean chirp duration was not solely explained by this finding. The following analyses were conducted using 3500 chirps from each of the sound recordings, showing that the described longer chirp duration (Fig. 4D) remained consistent when comparing 3-syllable chirps only (Fig. 6A; nested *t*-test, *P*<0.008) as well as when including 4-syllable chirps only in the analysis (Fig. 6B; nested *t*-test, *P*=0.022).

Hence, in addition to the difference in the relative proportion of syllables (3 versus 4), the significant difference in chirp duration may also result from one or both of the following: (i) differences in the duration of each syllable and (ii) differences in the duration of the inter-syllable intervals (for definitions, see Fig. 1). Interestingly, while the mean syllable duration did not differ between the LD and LL treatments (Fig. 6C,D; nested *t*-test, *P*>0.6), the mean duration of the inter-syllable interval was found to be significantly shorter under LL conditions compared with LD conditions for both the 3- and the 4-syllable chirps (Fig. 6E,F; nested *t*-test: *P*<0.004 and *P*<0.05, respectively). Notably, the magnitude of the difference in the mean inter-syllable interval was greater for the 3-syllable chirps than for the 4-syllable chirps.

### Female cricket preferences

We have established light-induced differences in male calling song properties. This, however, may be irrelevant for female mate choice. Therefore, in a preliminary set of experiments, we tested the possibility of the noted differences in male calling song properties affecting female choice (Fig. S1). LD females were simultaneously presented with a choice of two typical calling songs, that of an LD male and an LL male. The females displayed a significant preference towards the LD male song (Fig. S2, Table S3). These very preliminary data are presented in the Supplementary Materials and Methods.

## DISCUSSION

The present study comprises a large-scale analysis of a dataset of male cricket calling songs, followed by an in-depth investigation utilizing an innovative code for semi-automated detection of calling-song properties. The findings provide new insights into the potential effect of lifelong exposure to constant light on male cricket calling-song characteristics and may suggest some implications for female mate choice.

The major reported light-induced change is the significantly shorter chirp duration under LL than under LD illumination conditions. This result may indicate changes in one of the following (or a combination of them): (i) the proportion of 3- to 4-syllable chirps in the calling songs, (ii) the duration of each syllable or (iii) the duration of the inter-syllable intervals. Using the large-scale analysis and our semi-automated code, we found that (i) and (iii) were indeed affected by the different illumination treatments, while (ii) was not (Fig. 6). Furthermore, while the overall mean duration of the inter-chirp interval was found to differ significantly between the LD and LL treatments, a detailed examination of the nature and origin of the results led to the conclusion that this observed difference in inter-chirp interval duration did not arise from actual variation in the intervals themselves. Rather, it resulted from the significantly unequal proportions of 3- and 4-syllable chirps within each treatment (Fig. 5). Physiologically, one syllable ‘equals’ one closing movement of the cricket's wings. The syllable-related changes thus reflect modulation of the wing movements. The neuronal mechanisms underlying song production (and females' song detection) is not further discussed here, but has been previously extensively studied (Bennet-Clark, 1999; Huber, 1962; Huber et al., 1989; Libersat et al., 1994; Michelsen et al., 1994; Oldfield et al., 1986; Popov et al., 1976; Schöneich, 2020; Schöneich et al., 2015; Schöneich and Hedwig, 2010).

The significant effect of light on the proportion of 3- to 4-syllable chirps reported herein may have some evolutionary implications. Female *Acheta domesticus* crickets were shown to exhibit a preference for males producing a higher proportion (>79%) of 3-syllable chirps in their calling songs, consequently selecting for larger males (Stoffer and Walker, 2012). Variation in the proportions of the number of syllables per chirp were also described in two *G. bimaculatus* sub-populations. Males in these sub-populations exhibit chirps comprising either 3–4 (south Turkmenistan and Tajikistan) or 4–5 syllables (Caucasus) (Popov and Shuvalov, 1977). The reported light-related changes in the proportion of 3- and 4-syllables per chirp thus raise questions regarding the possible impacts on the evolutionary trajectory of the species following exposure to constant illumination.

The results presented here illustrate that certain calling-song traits are affected by lighting conditions and that these may potentially be discriminated by female crickets. Previous studies reported longer chirp duration and longer inter-syllable interval as a song characteristic preferred by female crickets (up to a certain extent; Bent and Hedwig, 2023; Popov and Shuvalov, 1977; Wagner, 1996). A neuronal circuit dedicated to male calling song pattern recognition was reported by Schöneich et al. (2015). These authors found specific neurons with inter-syllable interval-dependent activity: a distinct response was observed for inter-syllable intervals of 15–25 ms (see fig. 3 of Schöneich et al., 2015). The average inter-syllable interval duration of LL males in our study was 11.71 ms. Such calling songs may thus be less effective in activating such a circuit in the females. However, Grobe et al. (2012) describes that *G. bimaculatus* females reacted overall to chirp periods (the sum of the chirp and the following inter-chirp interval) of 200–500 ms, and best reacted to chirp periods of 300–400 ms and syllable periods (the sum of the syllable and the following inter-syllable interval) of a magnitude of 30–40 ms. The herein presented chirp and syllable period durations are indeed included in the described range, indicating that both LD and LL male stridulation may still evoke positive phonotaxis in the females. Nevertheless, short chirps (<100 ms) were reported to be at the threshold of the female response profile (see fig. 3A of Grobe et al., 2012), indicating a disadvantage for males with a much higher proportion of 3-syllable chirps as presented in this study in Figs 5 and 6A.

In accord with the above, female crickets in our preliminary tests reacted to both LD and LL males, though they demonstrated a clear preference towards the LD male calling song, characterized by significantly longer chirp duration, longer inter-syllable interval and a higher proportion of 4- to 3-syllable chirps compared with the LL male. Interestingly, while female crickets are assumed to perceive all changes in the calling-song properties, some male traits, and accordingly calling-song properties, may be more dominant than others in the females' decision, and thus in their reflection of the males' fitness (Hall and Robinson, 2021; von Helversen et al., 2004).

Similar to the presented effects on male calling song, females could also be affected by illumination conditions and may show plasticity in their responsiveness to certain mate choice parameters. We do not exclude the possibility that future, more thorough studies, including playback experiments with LL females, may result in additional insights, and may even indicate a male–female coupled response to constant light, similar to the temperature coupling reported in katydids (Symes et al., 2017). Signal-preference co-evolution has the potential to be followed by population differentiation and stepwise speciation (Grace and Shaw, 2011).

The effects of light on cricket behavior are manifested at different levels. For example, lifelong exposure to ALAN was reported to affect courtship behavior of both sexes in *Teleogryllus commodus* (but no effect was found on the males' calling-song properties; Botha et al., 2017). A delay in the initial walk of female *Teleogryllus* crickets towards calling-song playbacks has also been reported (Thompson et al., 2019). In *G. bimaculatus* crickets, ALAN induced both instantaneous behavioral effects and changes in the daily timing of stridulation, subsequently threatening population synchronization (Fergus and Shaw, 2013; Levy et al., 2021, 2023, 2024b; Loher, 1989; Sokolove, 1975). The findings presented here regarding the effects of light on the crickets' calling-song properties add to these previously reported effects of ALAN on these insects, and are particularly important in light of the growing global concerns regarding artificial light and its detrimental effects on insect well-being (Durrant et al., 2020; Levy et al., 2024b; Owens et al., 2020; Owens and Lewis, 2018).

In addition to light, other abiotic factors such as temperature and noise have been reported to affect cricket stridulation and courtship behavior. In particular, temperature was found to directly affect stridulation properties. Dolbear's law, for example, describes the relationship between the ambient temperature and the cricket's calling-song chirp rate (Dolbear, 1897). Similarly, a proportional increase in frequency with rising temperature was also observed in *G. bimaculatus* crickets both in the laboratory and in the natural environment (Doherty, 1985; Levy et al., 2024a,b). Several recent studies on the impact of noise pollution on crickets have described a masking effect due to noise interfering with the perception of acoustic signals, adding noise to the harmful effects of anthropogenic pollution (Duarte et al., 2019; Schmidt et al., 2014).

Previous research on calling-song properties has relied on manual or partial syllable measurements, leading to relatively limited sample sizes (e.g. Doherty, 1985; Simmons, 1988; Verburgt et al., 2011). The novel code developed and described here enabled semi-automation of the chirp and syllable detection process, thereby greatly increasing the sample sizes (3500–6700 chirps per sound file) and enabling the presented comprehensive in-depth analysis.

As the results presented in this study illustrate, intraspecific communication in crickets is complex, crucial for their reproductive success and not yet fully understood. At this stage, we have insufficient understanding of the mechanism behind the observed changes. Our results may be related to the fact that wing opening is an active muscular process, which can therefore be affected by the ambient conditions. The closing of the wings, responsible for generating the syllable itself, emerges from a passive ‘jumping back’ movement, and is therefore not affected by the ambient conditions. Further research is needed, possibly on the effects of light on the neuromechanism and its development under constant light, to fully understand the mechanism through which light affects calling-song parameters, and how this may further impact intraspecific communication and mate choice in these insects.

## Supplementary Material

10.1242/jexbio.249404_sup1 Supplementary information
