# Supplementary material for: Light alters calling-song characteristics in crickets
Source: J Exp Biol. 2025 Feb 25;228(4):JEB249404. doi: 10.1242/jeb.249404 (PMC11928050; doi:10.1242/jeb.249404)
Supplement: Supplementary information [file jexbio-228-249404-s1.pdf]

# Supplementary Materials and Methods

## Calling song properties analysis

**Table S1.** Normality tests and transformations conducted to achieve normal distribution on male crickets' calling-song Data.

| **Variable** | **Transformation** |
|---|---|
| **All sound files** | |
| **Total Chirps count** | no need |
| Total Syllables count | y = cos(y) |
| 3 Chirp Proportion | y = sqrt(y) |
| 4 Chirp Proportion | y = y squared |
| Chirp duration (ms) | y = y squared |
| Inter-chirp-interval (ms) | no need |
| Dominant frequency | no need |
| Inter-chirp-interval_3-3 (ms) | y = tan(y) |
| Inter-chirp-interval 3-4 (ms) | no need |
| Inter-chirp-interval 4-3 (ms) | no need |
| Inter-chirp-interval 4-4 (ms) | y = cos(y) |
| Temperature | no need |
| **3500 chirps per sound file** | |
| **3 Syllable chirps** | |
| Chirp duration (ms) | no need |
| Syllable duration (ms) | no need |
| Inter-syllable-interval (ms) | no need |
| **4 Syllable chirps** | |
| Chirp duration (ms) | no need |
| Syllable duration (ms) | no need |
| Inter-syllable-interval (ms) | y = cos(y) |

Journal of Experimental Biology • Supplementary information

**Table S2.** Mean inter-chirp intervals between similar chirps (3- to 3-, and 4- to 4-syllable chirps) and dissimilar chirps (3- to 4-, and 4- to 3-syllable chirps) in the calling songs of male crickets.

| Variable | LD Mean / % | LL Mean / % | p value |
|---|---|---|---|
| **Total Chirps count** | 5289 | 5614 | 0.1056 |
| Inter-chirp-interval_3-3 (ms) | 262.1 | 254.6 | 0.2537 |
| Inter-chirp-interval 3-4 (ms) | | | NA |
| Inter-chirp-interval 4-3 (ms) | 262.4 | 239.8 | ***0.0001*** |
| Inter-chirp-interval 4-4 (ms) | 292.1 | 278.6 | 0.4329 |

**Preliminary Mate choice experiments:**

**Experimental Procedure**

*Rearing conditions*

Experimental female crickets were reared under LD conditions only. The females were separated from the main colony at the last nymphal stage and kept individually until 7-11 days post adult molting, when they were used for the choice experiments.

*Calling-song manipulation and playback*

The full analysis and comparison of LD and LL calling songs suggested clear lighting regime-induced differences in calling song properties, especially in chirp duration. Hence, for the initial female choice playback experiments, a one-minute-long calling-song fragment was extracted from two chosen (extreme) recordings: the LD recording fragment comprised long, 141 ms chirps; while the LL fragment comprised short, 93 ms chirps.

For further female choice experiments the following manipulations were conducted on the calling-songs in Audacity® Cross-Platform Sound Editor (open source software, version 3.2.1):

(i) Creating calling songs consisting of either only four or only three syllables per chirp (notably the most prevalent syllables per chirp in *G. bimaculatus* crickets). Chirps were extracted from the LD calling songs and edited to create two new playbacks differing in chirp duration (Table S3; i a,b) using the Audacity® Cross-Platform Sound Editor. The intervals between chirps were kept constant and the recordings were calibrated to have a similar amplitude using RavenPro 1.5 (Cornell Lab of Ornithology, Ithaca). All edited recordings were played at 70 dB SPL (measured as peak at 10 cm distance at the speaker's location) and looped using RavenPro 1.5 to last three minutes each.

(ii) Creating new calling songs for both the LD and the LL groups, consisting of three syllables per chirp only. All 4-syllable chirps were excised from the LD and LL files, while keeping the 3-syllable chirps and their inter-chirp intervals untouched (Table S3; ii a,b). Recordings were again looped using RavenPro 1.5 to last three minutes each and played at 70 dB SPL (measured as peak at 10 cm distance at the speaker's location).

Journal of Experimental Biology • Supplementary information

*Experimental setup and procedure*

Mate choice experiments were conducted in a 182 cm X 265 cm arena. Two PiZZi Mini- DUO speakers (35x35x34mm, PZ-5700-6, Pizzi) were placed at the same distance of 120 cm and at an angle of 40° from the start point (Figure S1a, distance between speakers was 180 cm). During the experimental trials the room was kept at a temperature of 25±2°C, darkened, and lit only by a dim red light mimicking darkness for the cricket while enabling tracking and recording its movement by way of an infrared (IR) camera (2MP night-vision USB camera, emitting IR light, 170 degrees wide) installed on top of the arena. Video recording was conducted using the Bandicam Screen Recorder Software (version 6.0.4, NSH software, Greenwood, USA).

In each experiment, a single adult female cricket was placed under a perforated plastic cup in the arena and exposed to two different calling songs (of 70 dB SPL intensity), played simultaneously, each from one of the two speakers. Each speaker played a different randomly selected stimulus (e.g., LD or LL ($n = 16$), 3 vs. 4 chirps ($n = 39$), 3-chirps only LD vs. LL ($n = 22$). After one minute the cup was remotely lifted by pulling a string and the cricket could walk freely in the arena. The playbacks from both speakers lasted for an additional two minutes (three minutes in total). Experiments ended when a female made contact with one of the speakers (Figure S1b), hence selecting one song over the other, or at the end of the three minutes if no contact was made. Overall, females were tested up to three times. To ensure responsiveness and avoid habituation, the females participated in only one experiment per day. Between experiments, the arena floor was sprayed with 70% ethanol and wiped down with a clean rag. Each two trials were separated by an 8-minute interval. The female's choice, and the time elapsed until making contact with a speaker, were compared between treatments. Females, which did not make contact with any of both speaker, hence did not choose a male, were discarded from the analysis.

*Statistical analysis*

Female mate-choice comparisons and the time it took the female to reach the speaker after the cup was lifted (latency-to-choice) were conducted using a $\chi^2$ test and a Mann-Whitney test, respectively.

## Results

*The female cricket preferences*

Female crickets exposed simultaneously to the calling-song playbacks of an LD and an LL male exhibited a significant preference towards the LD individual (Chi-square test; $\chi_{1,16} = 5.24$, $p = 0.002$; Fig. S2a).

In order to determine whether *G. bimaculatus* female choice is affected by a preference for a specific number of syllables per chirp, females were exposed simultaneously to calling-song

Journal of Experimental Biology • Supplementary information

playbacks composed of either 4-syllable chirps or only 3-syllable chirps. This difference in the number of syllables per chirp seemed to be discerned by the females, which presented a perceptible, although non-significant trend towards the 4-syllable chirps (Chi-square test; $\chi_{1,38} = 2.63$, $p = 0.1$; Figure S2b). The latency-to-choice, i.e. the time until the female made contact with the chosen speaker, did not differ significantly between the two tested groups (Mann-Whitney test; $p = 0.58$, 20 s and 22.5 s for 3-syllable chirps and 4-syllable chirps, respectively).

As shown in Figure S2c, no female preference was shown (Chi-Square test, $\chi_{1,24} = 0.18$, $p = 0.67$) when testing the songs of LD or LL males that comprises only 3 syllables per chirp (after eliminating all 4-syllable chirps from the recordings, see Methods - manipulation ii, for details). In addition, no difference was observed in the latency-to-choice (Mann-Whitney test; $p = 0.76$).

Journal of Experimental Biology • Supplementary information

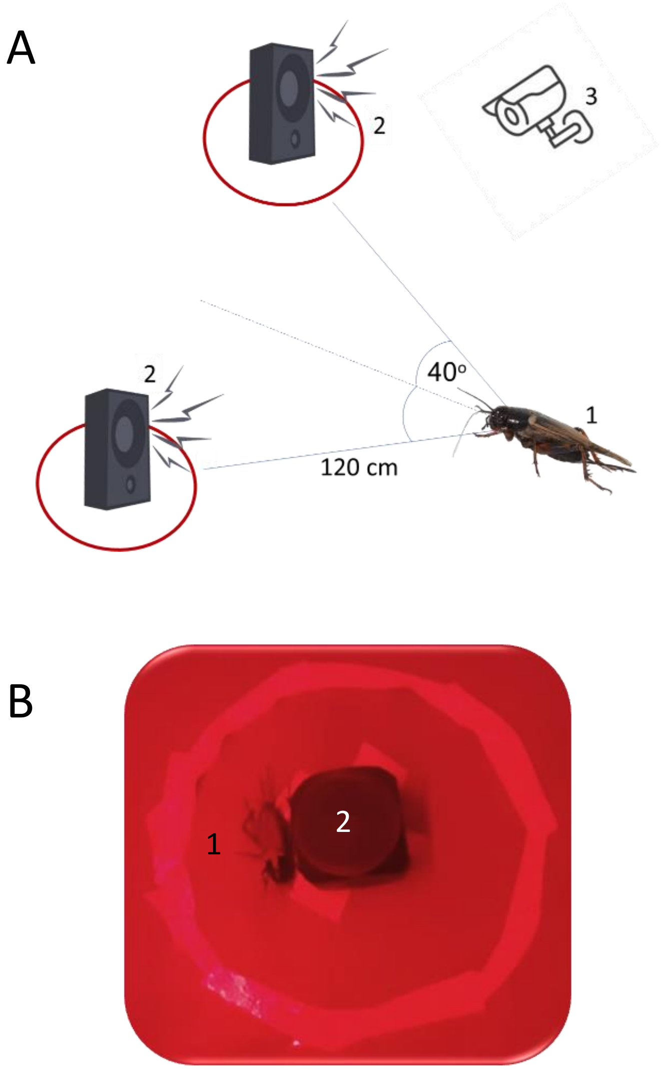


**Fig. S1.** The experimental setup. (A) a female cricket (1) is placed at a distance of 120 cm and a 40° angle from a right and left speaker (2) playing simultaneously two different stridulation recordings. The female's choice and trajectory are recorded by an infrared (IR) camera (3). (B) The female cricket (1) makes contact with the speaker (2), presenting the female's choice.

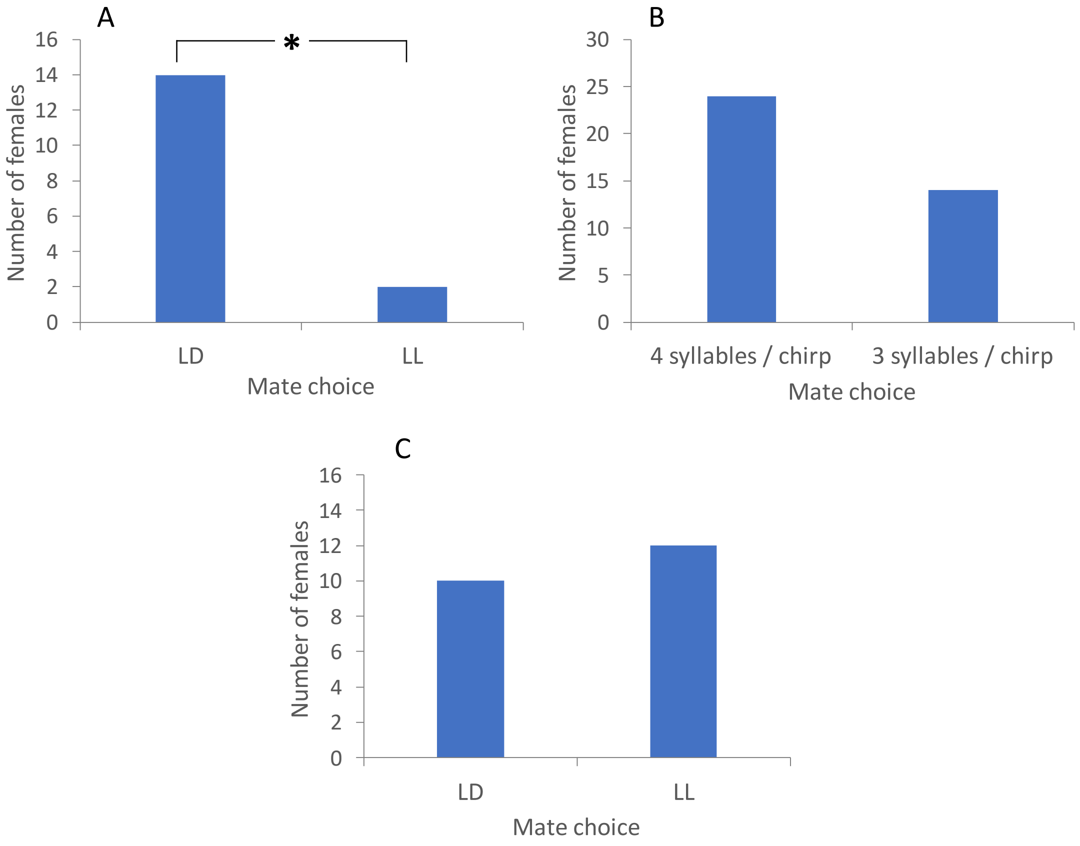


**Fig. S2.** Female crickets' choice experiments. (A) LD (n = 14) vs. LL (n=2) calling songs. $*p < 0.05$. (B) Four-syllable chirps (n = 24) vs. three-syllable chirps calling songs (n = 15). (C) LD vs. LL calling songs (n = 10, n = 12, respectively), both comprising three-syllable chirps only. The latter two, present no significant female preference.

Journal of Experimental Biology • Supplementary information

**Table S3.** Properties of the male cricket calling songs utilized in the playback experiments. i – Calling songs of male crickets, reared under lifelong light:dark (LD), manipulated to include either 3- or 4-syllable chirps only. ii - Calling songs of crickets reared under either LD or constant light (LL), manipulated to include 3-syllable chirps only.

| | | Rearing illumination condtions | Syllable / chirp | Chirp duration (ms) | Inter-chirp interval (ms) | Syllable duration (ms) | Inter-syllable interval (ms) | Fre-quency |
|---|---|---|---|---|---|---|---|---|
| i | a | LD | 3 Syllable chirps | 107.0 | 180 | 29.34 | 9.55 | 5115 |
| | b | LD | 4 Syllable chirps | 149.0 | 180 | 29.5 | 11.20 | 5129 |
| ii | a | LD | 3 Syllable chirps | 107.5 | 236.5 | 28.19 | 12.65 | 5081 |
| | b | LL | 3 Syllable chirps | 92.8 | 236.5 | 24.40 | 13.02 | 4909 |
